# An Adolescent Female With Concurrent Presentation of Autoimmune Hepatitis and Secondary Syphilis

**DOI:** 10.1097/PG9.0000000000000382

**Published:** 2023-11-27

**Authors:** Madison Riddell, Chris Novak, Sarah Dinn, Gary Galante

**Affiliations:** From the * Department of Pediatrics, Alberta Children’s Hospital, Cumming School of Medicine, University of Calgary, Calgary, Alberta, Canada

**Keywords:** gastroenterology, syphilis, pediatrics, autoimmune hepatitis, jaundice, hepatitis

## Abstract

We describe concurrent diagnoses of autoimmune hepatitis (AIH) and secondary syphilis in a 17-year-old adolescent with jaundice, with possible syphilitic hepatitis (SH) excluded after a thorough investigation. Our patient presented with a several-day history of malaise, progressive jaundice, and vomiting. She disclosed being sexually active and requested testing for sexually transmitted infections. Her subsequent investigations demonstrated acute hepatitis with a positive antinuclear antibody and elevated IgG. She also tested positive for syphilis with a reactive rapid plasma regain and treponema pallidum particle agglutination assay. We considered 2 etiologies for her elevated liver enzymes: syphilitic hepatitis and AIH. AIH was confirmed on liver biopsy, establishing the first reported pediatric case of concurrent AIH and secondary syphilis. Syphilis is hypothesized to be an infectious trigger for AIH.

## INTRODUCTION

The differential for jaundice presenting in the adolescent population is broad and includes infection, immunologic, structural, genetic, and drug-related causes. There may be vastly different etiologies for hepatitis considered based on historical features such as age, geographic location, and underlying medical conditions. Thus, in the workup of hepatitis, it is important to cast a wide net of investigations and consider the entire clinical picture to ensure all potential causes are taken into consideration and ruled out appropriately.

## CASE PRESENTATION

A 17-year-old previously healthy non-obese female presented to the emergency department with a 2-day history of progressive jaundice, scleral icterus, and vomiting, and 1 week of malaise and fatigue with intermittent headaches. She reported occasional marijuana use but denied other recreational drugs, medications, or alternative medicine. There was no personal or family history of autoimmunity, and routine immunizations were up-to-date. Physical examination revealed scleral icterus and jaundice without additional extrahepatic stigmata of liver disease and right-sided abdominal tenderness. Her initial investigations revealed markedly elevated transaminases in a mixed hepatocellular and cholestatic pattern, hypoalbuminemia, and elevated international normalized ratio not corrected with vitamin K (Table 1). Abdominal ultrasound was normal. She was admitted to the pediatric unit for further evaluation.

**TABLE 1.** Relevant investigations for etiology of mixed cholestasis-hepatitis in an adolescent

Investigation	Lab value (reference range)
Hemoglobin	138 (120–160 g/L)
MCV	87 (82–100 fL)
Platelet count	351 (150–400 10E9/L)
WBC	5.5 (4.0–11.0 10E9/L)
INR	**1.4** (0.9–1.1)
PTT	**42.2** (28–38 seconds)
Albumin	**28** (30–45 g/L)
Ammonia	31 (12–47 umol/L)
Bilirubin total	**201** (0–24 umol/L)
Bilirubin direct	**150** (0–7 umol/L)
ALP	**147** (50–140 U/L)
ALT	**807** (<= 34 U/L)
AST	**1125** (8-32 U/L)
GGT	**102** (<=26 U/L)
Lipase	21 (0–80 U/L)
CRP	**10.6** (0.0–8.0 mg/L)
COVID-19 NAAT test	Negative
Respiratory virus PCR panel	Negative
Gastroenteritis virus PCR panel	Negative
Parvovirus B19 IgM	Negative
*Chlamydia trachomatis* PCR test	Negative
*Neisseria gonorrhoeae* PCR test	Negative
HIV serology (mixed Ag/Ab detection)	Nonreactive
Hepatitis A antibody IgM	Negative
Hepatitis B surface antigen	Negative
Hepatitis C antibody	Nonreactive
Immunoglobulin A (0.60–4.20 g/L)	1.76
Antitissue transglutaminase (0.0–14.9 kIU/L)	<0.5
Immunoglobulin G (6.80–18.00 g/L)	**20.40**
Antismooth muscle antibody	Negative
Antinuclear antibody	**1:80, speckled pattern**
Antiliver kidney microsomal antibody	Negative
Thyroid-stimulating hormone (0.20–4.00 mIU/L)	2.59
Alpha-1 antitrypsin (0.90–2.00 g/L)	1.84
Ceruloplasmin (0.16–0.45 g/L)	0.22
Abdominal ultrasound with Doppler	Normal (no obstruction or signs of liver disease)

Bold values indicate abnormal values.

ALP = alkaline phosphatase; ALT = alanine aminotransferase; AST = aspartate aminotransferase; CRP = C-reactive protein; GGT = gamma-glutamyl transferase; HIV = human immunodeficiency virus; INR = international normalized ratio; MCV = mean corpuscular volume; NAAT = nucleic acid amplification Test; PCR = polymerase chain reaction; PTT = partial thromboplastin time; WBC = white blood count.

**FIGURE 1.**
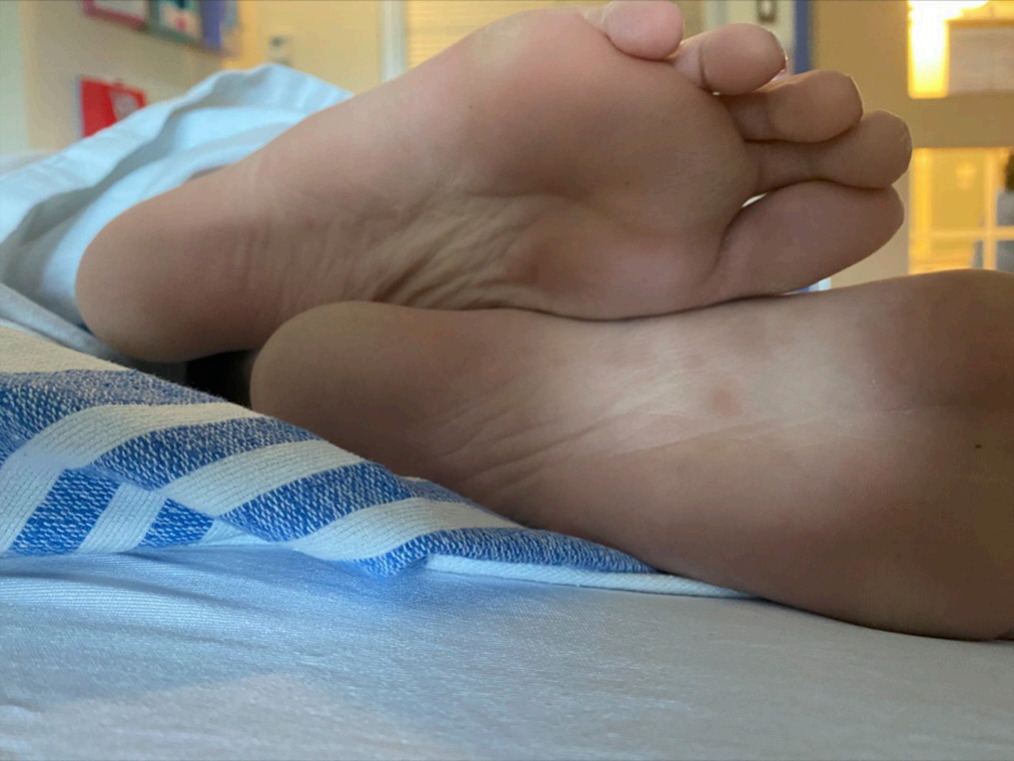
The rash that developed following antibiotics to the palms and soles of our patient, representing the Jarisch–Herxheimer reaction.

She disclosed being sexually active and that her current partner had recently tested positive for chlamydia. Therefore, in addition to the initial workup for cholestatic jaundice, she also requested testing for sexually transmitted infections (STIs). Incidentally, a urine culture collected in the emergency department grew *Escherichia coli*, and she was started on cefixime for a urinary tract infection. The following day, she developed a fever and a diffuse maculopapular, erythematous rash that was prominent on her palms and soles (Fig. 1). Further evaluation confirmed 2 new diagnoses.

The patient’s initial hepatitis workup was significant for positive antinuclear antibody and elevated IgG, without evidence supporting another etiology (Table 1). However, the patient’s STI testing revealed positive nontreponemal and treponemal tests for syphilis. Thus, concerns were raised for 2 possible causes for liver injury: autoimmune hepatitis (AIH) and secondary syphilis (SS).

**FIGURE 2.**
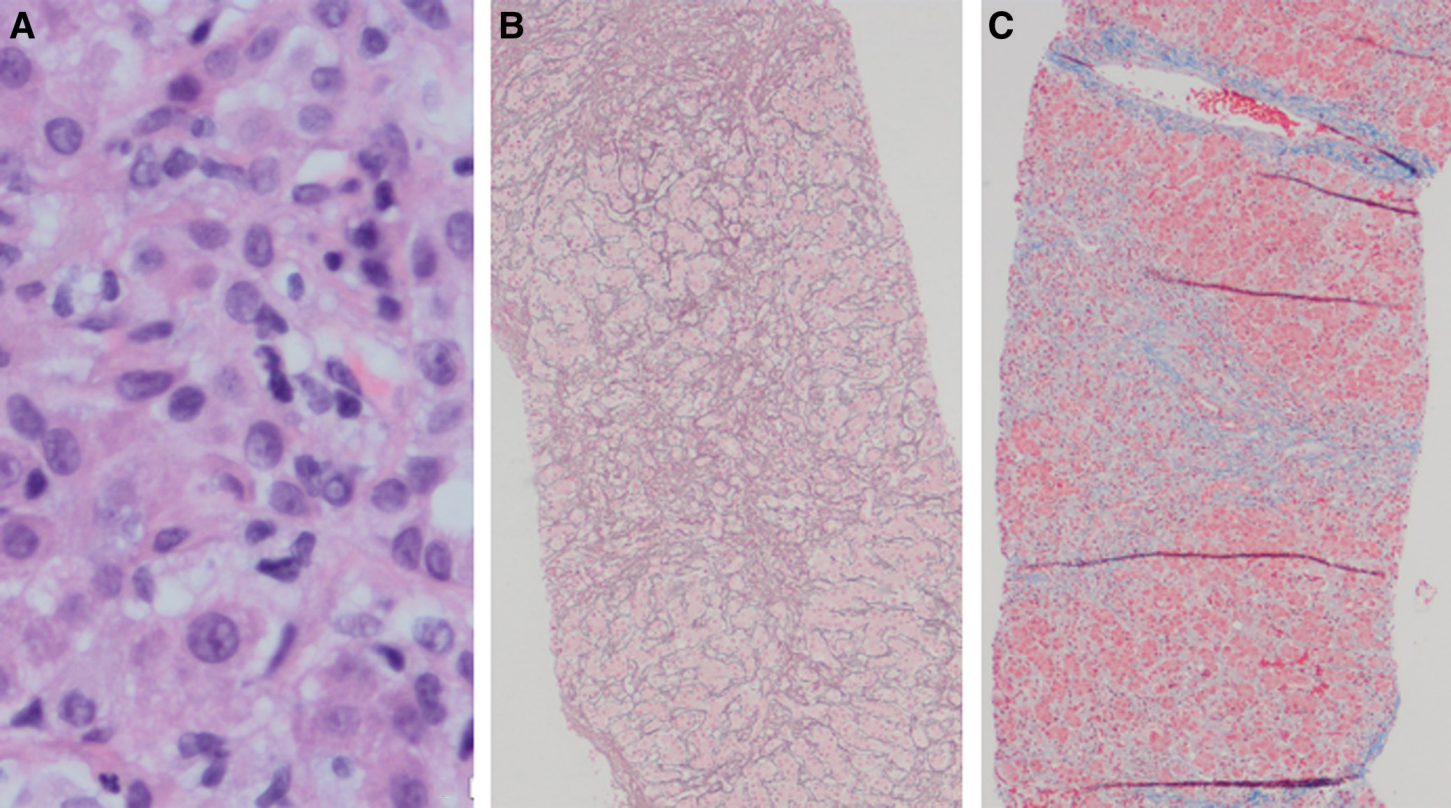
Liver biopsy with characteristic autoimmune hepatitis findings including (A) Hematoxylin and Eosin stain showing emperipolesis is present with intact lymphocytes within the cytoplasm of hepatocytes as well as multiple plasma cells typical of AIH, (B) Reticulin stain showing periportal and lobular areas of collapse of the reticulin network and (C) Masson Trichrome stain showing marked periportal and lobular pericellular fibrosis with early bridging fibrosis. No features of cirrhosis (eg, regenerative nodules and distorted hepatic architecture) are present. Photos provided courtesy of Dr. Weiming Yu.

The patient was treated for SS with a single dose of intramuscular penicillin G. Transaminases interestingly decreased following antibiotics with the aspartate aminotransferase decreasing by nearly 30% from a peak value of 959 U/L to 696 U/L following treatment, but given that her labs favored AIH, a liver biopsy was done to confirm a diagnosis and differentiate from syphilitic hepatitis (SH) (Fig. 2). Interface hepatitis (lymphoplasmacytic portal tract inflammation that invades surrounding parenchyma), the classic yet nonspecific histologic finding in AIH, was present (1). Biopsy additionally demonstrated hepatocyte rosetting and emperipolesis (internalized cells), also associated with AIH (1), and early bridging fibrosis. Further, no histologic features described in SH were noted, such as pericholangiolar inflammation, granulomas, periportal necrosis, or spirochetes (2), with negative immunostaining for *Treponema pallidum*.

Thus, our patient had concurrent diagnoses of SS and AIH-1, the first such reported pediatric case.

Following confirmation of AIH, she promptly received initial conventional immunosuppressive treatment with prednisone, typically recommended at 2 mg/kg/day (maximum 60 mg), to prevent disease progression (1). Transaminases and IgG normalized within 8 weeks of treatment. During this time, prednisone was gradually decreased, and azathioprine was added as a steroid-sparing agent as per our center’s protocol, supported by current recommendations (1).

## DISCUSSION

AIH exhibits female predominance, bimodal age of onset, with the first peak in childhood/adolescence, with type 1 (AIH-1) more common in pediatrics and generally presenting in adolescence. It is a progressive inflammatory hepatopathy involving loss of immune tolerance; while a specific cause remains unknown, pathogenesis likely involves both environmental and genetic factors. AIH may be associated with an infectious trigger (2). Presentation varies but mostly involves acute or insidious onset of nonspecific symptoms, such as malaise, nausea/vomiting, abdominal pain, and jaundice. Diagnosis is supported by positive autoantibodies and elevated IgG, with the exclusion of other liver disease (1). Positive autoantibodies, including antinuclear antibody, are an important initial diagnostic test in the diagnosis of AIH and help delineate type 1 and type 2 AIH, which can further inform the expected clinical course (1,3).

Thus, AIH was strongly suspected in our patient, but the contribution of syphilis was also considered, even though it may have been an incidental finding. SS rarely (3%) presents with SH, usually with disproportionate biochemical cholestasis relative to normal or mildly elevated transaminases (2), in distinction to AIH, characterized by transaminase elevation with or without cholestasis. SS is a difficult condition to diagnose given its varied nonspecific manifestations, including nonspecific constitutional symptoms as with our patient, lymphadenopathy, and characteristic rash involving the palms and soles. Further, it remains essential to complete a thorough social history in adolescent patients and maintain a low threshold for STI testing in this population, given that syphilis is one of several STIs that may cause hepatitis.

Interestingly, several symptoms of SS may be accentuated during the Jarisch–Herxheimer reaction, a treatment complication our patient likely developed after starting cefixime for urinary tract infection, which has incidentally shown efficacy in treating syphilis despite not being the preferred regimen. It usually occurs within 24 hours of starting antibiotic treatment for spirochete infections and presents with transient symptoms of fever, chills, nausea, flushing, and exacerbation of infection-related skin lesions. The pathophysiology underlying Jarisch–Herxheimer reaction is unknown but is postulated to be driven by the killing of spirochetes by antibiotics, with the subsequent release of cytokines and toxins initiating an acute inflammatory reaction.

The association of these 2 disease processes in this patient’s case prompted consideration of the possibility that syphilis infection may have triggered her AIH. Previous case reports (4) have implicated SH and its treatment in the activation of an immunologic cascade resulting in AIH. This link has been postulated through the phenomenon of molecular mimicry, whereby *Treponema pallidum* may activate the host immune system against its own tissues (2). *T. pallidum* affects innate and adaptive immune responses. However, further research is needed to confirm *T. pallidum* as a possible trigger for autoimmune conditions.

## ACKNOWLEDGMENTS

This report was written after informed consent was obtained and documented from the patient and confirmed in clinic after she turned 18.
